# A test for clinal variation in *Artemisia californica* and associated arthropod responses to nitrogen addition

**DOI:** 10.1371/journal.pone.0191997

**Published:** 2018-02-01

**Authors:** Maria M. Meza-Lopez, Kailen A. Mooney, Amanda L. Thompson, Nicole K. Ho, Jessica D. Pratt

**Affiliations:** Department of Ecology and Evolutionary Biology, University of California, Irvine, Irvine, California, United States of America; Washington University, UNITED STATES

## Abstract

The response of plant traits to global change is of fundamental importance to understanding anthropogenic impacts on natural systems. Nevertheless, little is known about plant genetic variation in such responses or the indirect effect of environmental change on higher trophic levels. In a three-year common garden experiment, we grew the shrub *Artemisia californica* from five populations sourced along a 700 km latitudinal gradient under ambient and nitrogen (N) addition (20 kg N ha^-1^) and measured plant traits and associated arthropods. N addition increased plant biomass to a similar extent among all populations. In contrast, N addition effects on most other plant traits varied among plant populations; N addition reduced specific leaf area and leaf percent N and increased carbon to nitrogen ratios in the two northern populations, but had the opposite or no effect on the three southern populations. N addition increased arthropod abundance to a similar extent among all populations in parallel with an increase in plant biomass, suggesting that N addition did not alter plant resistance to herbivores. N addition had no effect on arthropod diversity, richness, or evenness. In summary, genetic variation among *A*. *californica* populations mediated leaf-trait responses to N addition, but positive direct effects of N addition on plant biomass and indirect effects on arthropod abundance were consistent among all populations.

## Introduction

Many plant species span broad environmental gradients and, as a result, can adapt to different abiotic and biotic conditions with consequences for plant-associated communities [1]. Environmental gradients can thus lead to genetically-based clinal variation in ecologically important plant traits [2–4] including adaptive plasticity in those traits [5]. Genetic variation in plant traits can in turn influence interactions with other community members [6,7]. If plant genetic variation is clinal, it could lead to clinal variation in plant-associated communities [8].

Plant traits and associated communities are driven not only by plant genetics, but also by the plastic response of plants to changing abiotic conditions, including those associated with global change [9–12]. Changing abiotic conditions can have direct effects on plants, but such effects can also propagate to indirectly influence plant-associated communities [13]. In addition, the magnitude of plant plastic response to changing abiotic conditions may vary across a species range [8] and, as a result, plant-associated communities may likewise vary with changing abiotic conditions.

Atmospheric nitrogen (N) deposition is one driver of global change that can significantly impact biological communities [6,14]. N deposition tends to increase foliar N concentrations, determining plant quality and defensive chemistry, which can affect herbivore survival, growth, and reproduction [15,16]. For instance, N addition increased N tissue content in *Brassica oleracea*, indirectly increasing diamondback moth populations (*Plutella xylostella*) [17]. As a consequence, the direct effects of N deposition on plant traits may indirectly influence plant-associated communities both above and below ground, as has been shown for plant-associated arthropods [15,18] and soil microbial communities [18,19], whose direct responses to N can also mediate plant responses [18–20]. Although the consequences of N deposition for plants and associated communities have received some attention [15,21], the importance of intraspecific variation in plant responses to N deposition and the consequences for plant-associated communities are unknown.

In this study, we investigated the role of *Artemisia californica* intraspecific variation in response to N deposition and its indirect effects on associated arthropod communities. A past study of five *A*. *californica* populations sourced along a 700 km latitudinal gradient under ambient and increased (4-fold) precipitation revealed clinal variation in plant growth, leaf-traits, and plasticity [5,22], resulting in clinal variation in arthropod community composition [8]. Plants from southern (vs. northern) populations had higher growth rates, carbon to nitrogen ratios, and terpene concentrations with lower specific leaf area, leaf percent N [5,22], and arthropod density [8]. In addition, plants from southern (vs. northern) populations exhibited greater plasticity in growth and flowering in response to water addition [5].

Here, we characterize intraspecific variation in *A*. *californica* traits in response to N deposition and the indirect effects of N deposition on associated arthropod communities. Specifically, we grew plants sourced from 5 populations along the California coast in a single common garden under ambient conditions and N addition to simulate regional atmospheric N deposition. In so doing, we addressed the following questions: *(i)* How does *A*. *californica*’s genetic variation influence growth and functional traits?; *(ii)* how does *A*. *californica* respond to N deposition and is there population variation in such responses?; and *(iii)* how does N deposition affect associated arthropod communities across these plant populations? In addressing these questions, this study increases our understanding of the direct effects of N deposition on plants and its indirect effects on associated arthropod communities, while evaluating how plant genetic variation influences these responses.

## Materials and methods

### Study system

California sagebrush (*Artemisia californica* Less., Asteraceae) is a foundation plant species in Coastal Sage Scrub habitat, a low elevation scrubland plant community that is considered one of the most threatened communities in the United States [23,24]. *Artemisia californica* is a long-lived drought-deciduous shrub endemic to California with a range extending from Baja, Mexico to Mendocino County, CA. Furthermore, coastal sage scrub vegetation is highly fragmented due to land-use change and development, and has been reduced to 10–15% of its historical distribution [23,24]. Additionally, *A*. *californica* supports a diverse arthropod community (>230 spp.) [5,8,24,25], which is the primary food source for many endemic and endangered vertebrate species, including the California gnatcatcher (*Polioptila californica*), coastal cactus wren (*Campylorhynchus brunneicapillus* subsp. *couesi*), orange-throated whiptail (*Aspidoscelis hyperythra*), and the San Diego horned lizard (*Phrynosoma coronatum* subsp. *blainvillii*).

### Experimental protocols

#### Common garden

In April 2009, we collected 20 plant cuttings from 10 *A*. *californica* plants (N = 200 cuttings per population) from each of five source populations (Scripps Coastal Reserve, San Diego, CA [32°52’26.89” N, 117°14’54.87” W; 95 m a.s.l.; hereafter ‘SD’], Santa Monica Mountains National Recreation Area, Santa Monica, CA [34°03’54.09” N, 118°59’12.83” W; 80 m a.s.l.; hereafter ‘SM’], Kenneth S. Norris Rancho Marino Reserve, Cambria, CA [35°31’39.92” N, 121°04’26.5” W; 20 m a.s.l.; hereafter, ‘CAM’], Wilder Ranch State Park, Santa Cruz, CA [36°58’12.22” N, 122°07’22.74” W; 20 m a.s.l.; hereafter, ‘SC’], and Rodeo Beach, Golden Gate National Recreation Area, San Francisco, CA [37°50’1.95” N, 122°32’40.53” W; 57 m a.s.l.; hereafter, ‘GG’]), representing 70 percent of its latitudinal range. Permission to collect cuttings of *A*. *californica* was granted from the United Stated Department of the Interior’s National Park Service (sites SM and GG; permit SAMO-2008-SCI-0003), from the University of California’s Natural Reserve System (sites SD and CAM), and from the overseeing Park Ranger at Wilder Ranch State Park (site SC). Table 1 shows estimated total and dry N deposition at each of these sites obtained from 2011 total deposition (TDep) model runs, a measurement-model fusion incorporating empirical data to modeled data from the Community Multiscale Air Quality (CMAQ) model estimates [26].

**Table 1:** Estimated total and dry N deposition (kg N ha^-1^ yr^-1^) in 2009.

Location	Site	Total N	Dry N
Newport Beach	Upper Newport Bay Ecological Preserve	13.44	13.00
San Diego	Scripps Coastal Reserve	3.82	3.25
Santa Monica	Santa Monica Mountains National Rec Area	10.40	9.43
Cambria	Kenneth S. Norris Rancho Marino Reserve	4.46	3.95
Santa Cruz	Wilder Ranch State Park	6.50	5.55
San Francisco	Rodeo Beach, Golden Gate National Rec Area	7.84	5.69

Estimated total and dry N deposition where the common garden was located (gray) and at each of the 5 sites where cuttings were collected (from south to north).

Adult plants (~1 m^3^) from each population were haphazardly selected with the constraint that they were a minimum of 5 meters from one another and from large populations (i.e. at least a few hundred individuals). Populations were 100–500 m from the coast and at least 100 km apart from each other. Immediately after plant cutting collection, cut ends were wrapped with wet paper, placed in plastic bags, and stored in a cooler for transport to University of California, Irvine. Within 12–72 hours of collection, plant cuttings were dipped in a 20% solution of Dip ‘N Grow Root Inducing Concentrate (Dip ‘N Grow Inc., Clackamas, OR) and planted in horticultural perlite for 6 weeks. Approximately 80% of cuttings died, primarily due to fungal attack. This level of mortality for cuttings is similar to what we have found in previous experiments with *A*. *californica*. Rooted cuttings were transplanted to individual pots containing a soil mixture of equal parts silica sand, redwood compost, peat moss, and pumice, and grown in an outdoor shade house at the UC Irvine Arboretum for 10 months.

In March 2010, five plants of similar size (one from each population if available) were randomly selected and planted into 15 plots (75 plants total) in a common garden at the Upper Newport Bay Ecological Preserve in Newport Beach, CA (33°39’13.82” N, 117°53’5.8” W; 16 m a.s.l.) with the permission of the Orange County Parks who also provided logistical support. To minimize non-genetic (maternal-like) effects associated with plants cloned from cuttings [27], plants were grown in the greenhouse and field for a total of 24 months before measuring plant traits. The common garden consisted of seven blocks, each containing a pair of 2 x 2 m plots and an additional unpaired plot for a total of 15 plots with 2 m between plots and blocks. Plants from each population were evenly distributed among and randomized within the 15 plots. One plant from each of the five populations was included in 9 of the 15 plots and in 4 out of the 7 blocks. Due to high mortality of plant cuttings from fungal attack, GG plants (northernmost population) were substituted with plants from SD or SM populations in three blocks to maintain a consistent planting density in each plot. We speculate that cuttings from GG experienced higher fungal infection because they were in transport conditions the longest prior to transplant as this site required the longest distance travel. The number of replicate plants per population varied (SD = 19, SM = 17, CAM = 15, SC = 15, and GG = 9 replicates) with each population being represented by 10 separate genotypes (maternal source plants) in all cases except GG which was represented by 9 genotypes. Plants were watered through September 2010 to ensure establishment and survival during the seasonally dry summer.

The preserve is a degraded patch of upland habitat approximately 100 m from Newport Bay and 6 km inland from the ocean coastline with an estimated background N deposition of ~13 kg N ha^-1^ yr^-1^ in 2009 based on empirical deposition values from the National Atmospheric Deposition Program. At the time of the experiment, the site was covered by a mix of non-native grasses and forbs with a few native shrubs interspersed. Intact coastal sage scrub habitat was found in patches throughout the areas adjacent to the common garden. Additionally, we planted this common garden within 15 m of another common garden planted the previous year with plots containing *A*. *californica* from the same five source populations [5,8,22]. These two common gardens were planted independent of each other and designed to answer different research questions. Cuttings for this common garden and the previous year common garden came from the same locations, but cuttings were collected in different years and from different source plants. Finally, replication per population in this experiment was lower than the year prior common garden even though the same populations (cuttings were collected in different years for this and the year prior common garden) were used. The resultant trait data (described below) were more variable than in our previous studies, possibly due to the smaller sample sizes as well as abiotic conditions that differed between the two studies (e.g. slightly different planting locations, drought conditions).

We added N to one plot in each block using a two-step approach [28] to simulate available N for plant use in the natural cycle of N deposition in southern California. N deposition of 20 kg N ha^-1^ yr^-1^ is within the range of naturally occurring N deposition [29] and is 2x the estimated ‘critical load’ (of 10 kg N ha^-1^) for nitrogen-poor or low biomass vegetation in coastal sage scrub in southern California, which is at risk of major vegetation change. To achieve N deposition of 20 kg N ha^-1^ yr^-1^ for two growing seasons, we used a two-step N addition approach. First, we added “fast release” N in the form of Ca(NO_3_)_2_ in October 2010 and 2011. Then in December 2010 and 2011, we added “slow release” N (Florikan, 12% N by weight). This two-step N addition approach simulates the natural cycle of N deposition in southern California chaparral and coastal sage scrub ecosystems, where large N inputs from dry deposition accumulate on plants and soil surface during the dry summer and a large nitrogen input occurs with the first rains in winter that carry accumulated dry N on plants and soil surface followed by sustained N input from ongoing wet N deposition during the wet season until the next dry season when dry N builds up again [28,30]. To simulate the large N pulse that occurs with the first rain, we added fast release fertilizer; to simulate the sustained N input that occurs due to new deposition during the wet season, we added slow release fertilizer.

#### Plant growth and traits

We measured plant size three times annually from April 2010 –May 2012. To do so, we estimated plant biomass by quantifying the size of the plant in relation to a cut branch taken from a non-experimental *A*. *californica* plant. The estimate used was an average taken from two independent assessments of plant size. The cut branch was then dried at 60°C for 72 hours and weighed. This dry biomass weight was then multiplied by the average estimate for plant size in relation to the cutting to obtain a final estimate of dry biomass (g) for the living plant. We used estimated plant biomass in April 2011 as our estimate of growth because all plants began as cuttings of the same size and this measurement corresponded to the time when all other plant traits were measured.

We measured specific leaf area (SLA), percent N, carbon to nitrogen (C: N) ratios, and percent water content (PWC) once in March 2011 during peak growing season. We haphazardly collected 20 fully expanded leaves from the crown of each plant that represented the current year’s growth. Ten leaves were used to assess SLA and PWC and the other 10 leaves were used for leaf elemental analysis. To minimize potential mass loss, leaves were collected in the early morning and freshly picked leaves were immediately placed in envelopes on ice and kept cool until they were scanned and weighed (wet weight) within a few hours of collection that same day. Leaf area (cm^2^) was measured from scanned images using ImageJ software [31]. Leaves were then dried at 60°C for 72 hours and weighed again (dry weight), allowing us to calculate SLA as cm^2^g^-1^ dry weight and PWC as ((wet weight-dry weight)/wet weight). To assess leaf C and N elemental composition (for N content and C: N ratios), dried leaves were ground to a fine powder using a Wig-L-bug grinding mill (International Crystal Laboratories, Garfield, NJ). Approximately 1 mg of this homogenized powder was then packed into 5 x 9 mm tins for elemental analysis (Fisons Instruments 1500) that was then performed at the UC-Irvine Mass Spectrometry Facility.

#### Associated arthropod community

We sampled whole arthropod communities in May 2012 for each plant. To sample arthropods, we vacuumed each plant exhaustively with an electric shop vacuum (3.5 HP Rigid model #WD0970) for up to 2 minutes for the largest plants (range: 24–120 seconds). We collected arthropods into fine mesh bags inserted into the vacuum nozzle, placed them on ice immediately after collection, and transferred them to a -20°C freezer later that day. We stored arthropods in 70% ethanol after separating them from plant chaff and subsequently identified them to morphospecies within family [8,32,33] and to the genus or species level when possible for the most abundant arthropods. We assigned each morphospecies to one of nine guilds based on published accounts for taxonomic groups as described in Pratt et al. [8].

### Data analyses

We tested for main and interactive effects of population and N addition on plant growth, SLA, percent N, C: N ratios, and PWC, as well as arthropod abundance (individuals*plant^-3^), density (individuals*m^-3^ plant biomass), morphospecies richness, Shannon-Weiner Diversity (*H’*), and Pielou’s Evenness (*J’*). Richness, *H'* and *J'* were calculated in Primer 6 [34] using abundance data of adult morphospecies only. A significant population effect indicates genetic variation among populations, a significant N addition effect indicates trait plasticity in response to N addition, and a significant population x N addition interaction indicates genetic variation among populations in the degree of plasticity in response to N addition. All analyses were conducted using the MIXED procedure in SAS Version 9.2 [35], specifying block and nitrogen-by-plot interaction as random effects. To meet ANOVA assumptions of normally distributed residuals and homogeneity of variances, plant biomass was square root transformed and percent N and arthropod density were log-transformed while all other variables were untransformed.

## Results

**Fig 1:**
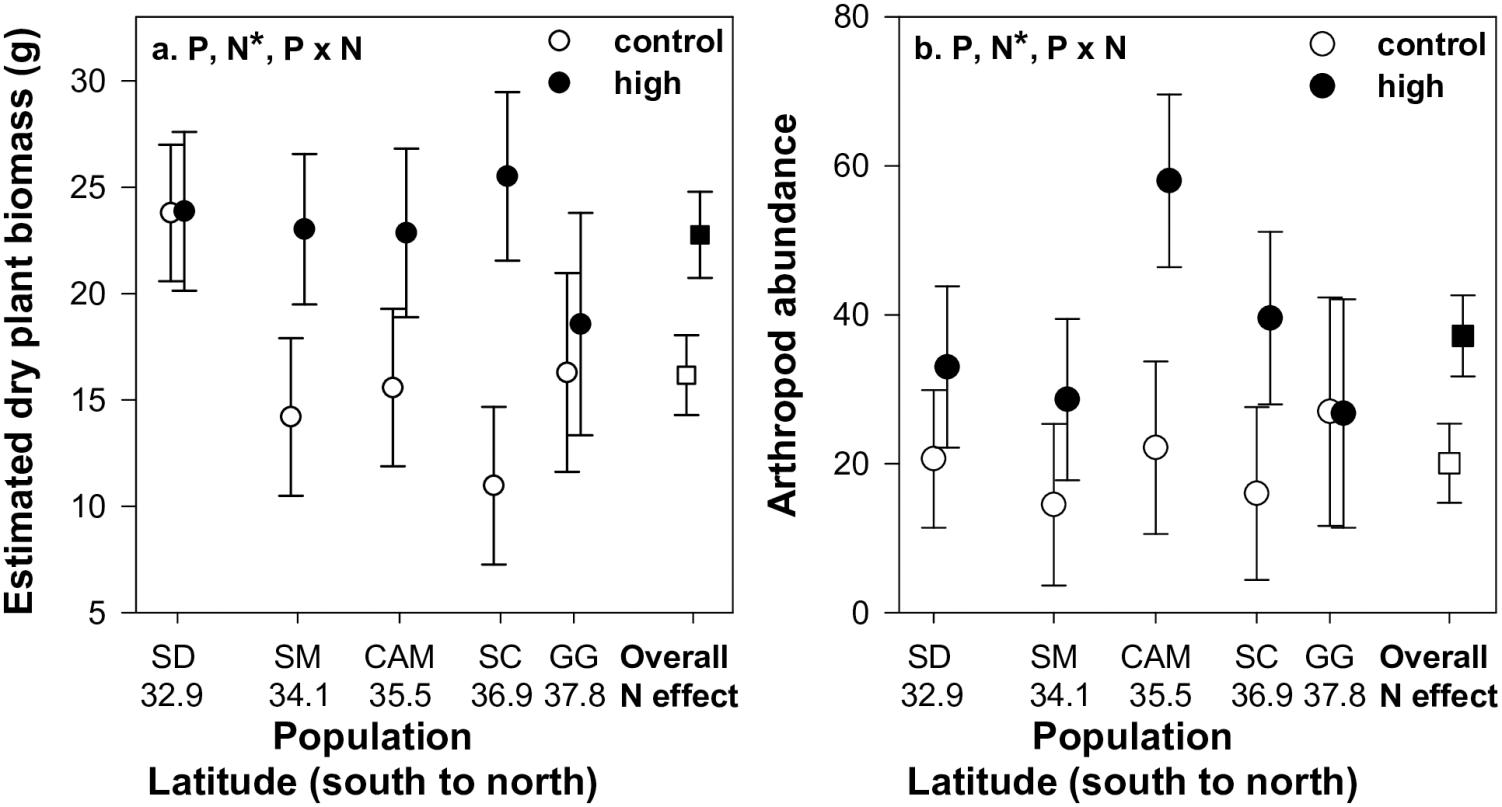
Plant population and nitrogen addition effects on plant biomass and arthropods. Main N addition effect (right side of each plot; squares) and interactive population and N addition effect (left side of each plot; circles) on a) estimated dry plant biomass (g) and b) arthropod abundance (individuals per plant). Letters represent populations: SD = San Diego, SM = Santa Monica, CAM = Cambria, SC = Santa Cruz, and GG = Golden Gate National Recreation Area. Numbers below letters represent population latitude. Error bars represent ±1SE. Treatments (P = plant population, N = nitrogen addition, and P x N = plant population x nitrogen addition) are listed at the top of each panel with (*) designating statistical significance.

**Fig 2:**
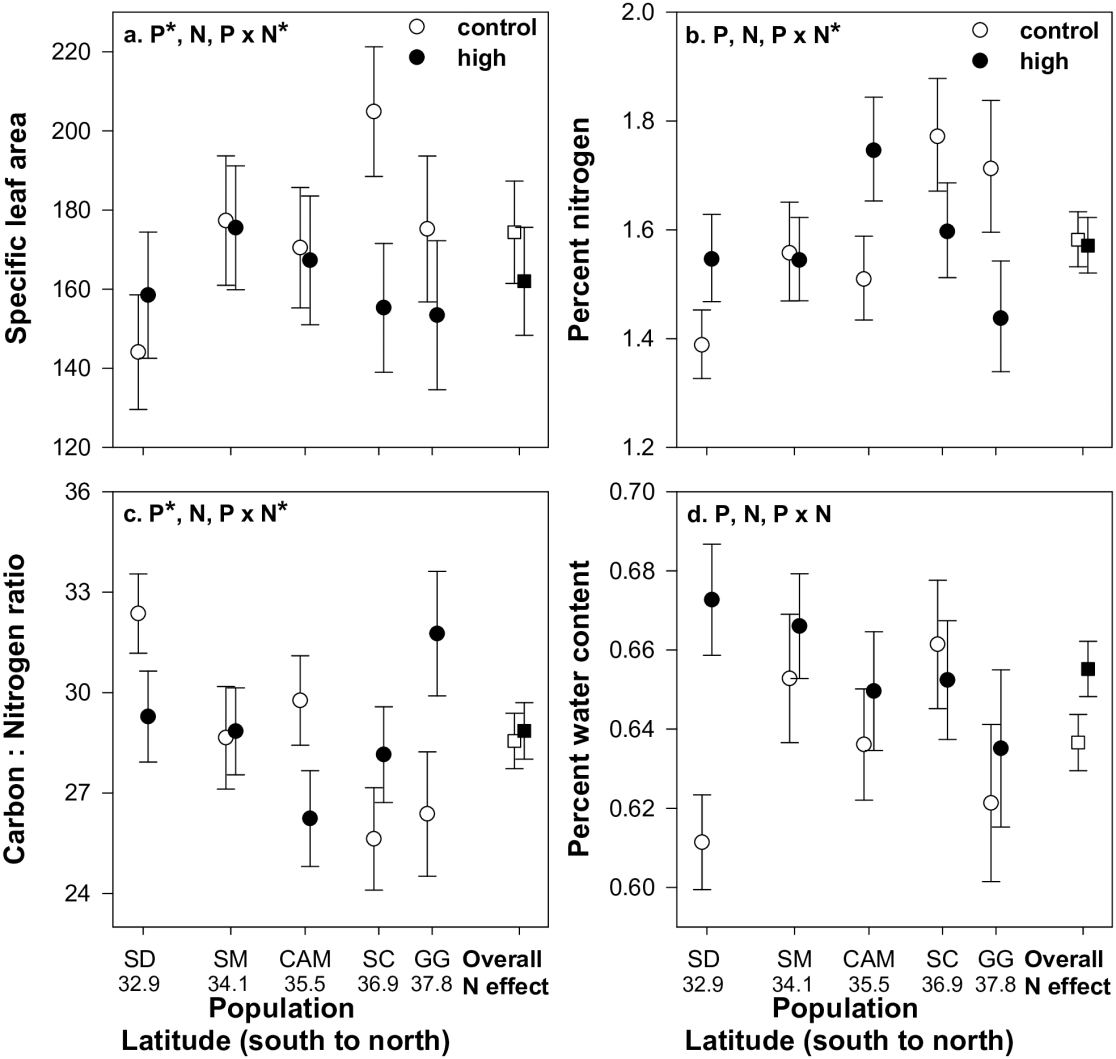
Plant population and nitrogen addition effects on plant traits. Main N addition effect (right side of each plot; squares) and interactive population x N addition effect (left side of each plot; circles) on a) specific leaf area (cm^2^g^-1^ dry weight), b) percent N, c) carbon to nitrogen ratio, and d) percent water content ((wet weight-dry weight)/wet weight). Letters represent populations: SD = San Diego, SM = Santa Monica, CAM = Cambria, SC = Santa Cruz, and GG = Golden Gate National Recreation Area. Numbers below letters represent population latitude. Error bars represent ±1SE. Treatments (P = plant population, N = nitrogen addition, and P x N = plant population x nitrogen addition) are listed at the top of each panel with (*) designating statistical significance.

N addition increased plant biomass by 41% among all populations with no main or interactive effect of population (Table 2 and Fig 1a). *Artemisia californica* populations varied in SLA and C: N ratios and plant populations with N addition influenced SLA, percent N, and C: N ratios (i.e. significant population x N addition; Table 2 and Fig 2). SLA values were on average higher and much more variable in this experiment than in our previous work, possibly due to differences between the two gardens resulting in different leaf phenology during our sampling timeframe (see Methods). N addition reduced SLA and percent N and increased C: N ratios for the two northern populations and had an opposite or no effect on these plant traits for the three southern populations (Fig 2). There were no main or interactive effect of population on leaf PWC but N addition alone increased plant biomass (Table 2 and Fig 2).

**Table 2:** Nitrogen addition and population effects on plants and arthropods.

Variable	Population	Nitrogen	Population x Nitrogen
	F_*DF*_ (P-value)	F_*1*,*13*_ (P-value)	F_*DF*_ (P-value)
Estimated plant biomass	0.99_*4*,*52*_ (0.4187)	**5.70 (0.0329)**	1.20_*4*,*52*_ (0.3238)
Specific leaf area	**2.86**_***4*,*47***_ **(0.0336)**	0.43 (0.5214)	**2.86**_***4*,*47***_ **(0.0335)**
Percent nitrogen	2.42_*4*,*47*_ (0.0612)	0.02 (0.8826)	**3.14**_***4*,*47***_ **(0.0227)**
Carbon to nitrogen ratio	**2.76**_***4*,*47***_ **(0.0386)**	0.06 (0.8043)	**3.34**_***4*,*47***_ **(0.0174)**
Percent water content	1.12_*4*,*47*_ (0.3607)	3.47 (0.0851)	1.74_*4*,*47*_ (0.1580)
Arthropod abundance	0.72_*4*,*48*_ (0.5803)	**5.07 (0.0423)**	0.56_*4*,*48*_ (0.6952)

Dependence of plant growth and traits and arthropod abundance on plant population, nitrogen addition, and the interaction of population and nitrogen addition. Significant results are shown in bold

N addition increased arthropod abundance but there was no main or interactive effect of plant population on arthropod abundance (Table 2 and Fig 2). Arthropod density, species richness, Pielou’s Evenness (*J'*), and Shannon-Wiener diversity (*H'*) did not depend on the main or interactive effect of plant population and N addition (all P>0.05; S1 Table and S1 Fig).

All data is available in S1 Dataset.

## Discussion

Studying a fundamental component of global environmental change, we demonstrated population variation in plant responses to N deposition with consistent indirect effects on associated arthropod communities. N addition effects on most plant leaf-traits varied which could be evidence of clinal variation among plant populations. N addition reduced SLA and leaf percent N and increased C: N ratios in the two northern populations and had an opposite or no effect on these traits in the three southern populations. In contrast, N addition increased plant biomass to a similar extent among all populations with parallel increases in arthropod abundance, such that N addition did not alter plant resistance to herbivores (i.e. arthropod density). Additionally, there were no N addition effects on arthropod richness, evenness, and diversity.

N addition increased plant growth, but these effects were not contingent upon plant population. N addition typically increases plant growth [36–39], but can have an opposite effect in areas with high N deposition [40]. For example, Allen et al. [40] found that N addition (high N deposition exceeding 50 μg N g^-1^ soil) reduced *A*. *californica* growth and survival at sites with high N deposition, but increased *A*. *californica* growth at sites with low N deposition, which is consistent with our study where we added 2 μg N g^-1^ soil at a low N deposition site. Furthermore, N addition effects on plant performance could also be influenced by other abiotic conditions such as water resources. Valliere et al. [41] suggested that N deposition under drought conditions increases plant mortality by increasing leaf area and reducing water use efficiency. Similarly, N deposition initially increased *A*. *californica* and then decreased performance and increased *A*. *californica* mortality under drought conditions, suggesting that shrub responses to N input can depend on its interactions with other abiotic conditions as well as species responses [42].

In our one-year study, we did not quantify temporal variation in plant traits response to N addition as has been done elsewhere [41]. Valliere *et al* [41] showed that high N deposition effects on shrub growth and productivity varied over time with leaf litter and leaf area peaking during the first year and declining in subsequent years. This is consistent with the large growth response that we observed in our study with N addition. Additionally, in their study, leaf tissue N increased with N addition over time while C: N decreased and was consistently lower in control plants while SLA varied with N addition with no consistent pattern [41]. Similarly, Vourlitis and Fernandez [43] found temporal variation in coastal sage scrub tissue N and C: N ratio characterized by increasing and decreasing differences between N addition and control treatments over time, respectively. With our data, we obtained a snap shot of N addition effects on plant traits and associated arthropod communities. This limits our ability to predict how plant traits and associated arthropod communities respond to N addition over time especially if N addition effects are influenced by other abiotic conditions (i.e. drought) that can also vary among years.

Our finding of no population variation in growth plasticity in response to N addition contrasts with our past work that demonstrated population variation in growth plasticity in response to precipitation [5]. We previously found population variation in *A*. *californica* growth and flowering in response to a precipitation manipulation of ambient southern precipitation vs. a four-fold increase in precipitation to simulate the northern precipitation regime. Studying these same 5 populations at an adjacent field site, growth and flowering were higher for southern than for northern populations [5]. The magnitude of population plasticity (i.e. increased growth and flowering in response to water addition) was closely correlated with interannual variation in precipitation, thus suggesting local adaptation in plasticity to variation in the coastal precipitation regime.

To our knowledge there is no temporal variation but there is spatial variation among populations in their exposure to N deposition that might drive population variation in plasticity in response to N addition. Total and dry N deposition increases from south to north except for the SM population with the highest exposure to N deposition compared to the other populations. In contrast to our past study [5], there was no clinal variation in *A*. *californica* growth under ambient N. Variation among populations in their exposure to N deposition could influence population variation in plasticity in response to N addition based on local adaptation, but we may not have been able to detect it possibly because N addition effects on plant growth may have been influenced by other abiotic conditions (i.e. drought) during our study. Dry or wet years may have also influenced population variation in response to N deposition. This may have been also due to other factors, including a smaller sample size (i.e. lower statistical power) compared to the year prior common garden and conducting this common garden on a site where plant growth was lower overall. Additionally, the timing was different for this common garden compared to the year prior common garden and this common garden was also conducted during drier and less productive years.

In contrast to plant growth, plant populations varied in leaf-traits with N addition suggesting that population variation could be clinal. We speculate that these patterns in leaf-trait responses to N addition could be due to underlying clinal variation in constitutive leaf-traits (i.e. traits under ambient N). In the absence of N addition, two northern populations (vs. southern populations) were characterized by high SLA and percent N and low C: N ratio, findings that are consistent with our past work on these same populations [5]. N addition in the two northern populations decreased SLA and percent N and increased C: N ratios. In contrast, the three southern populations were characterized by opposing constitutive trait values (lower SLA and percent N and higher C: N ratios) and responded oppositely (or did not change) with N addition (increasing SLA and percent N and reducing C: N ratio values or no change).

Variation in how southern and northern populations respond to N addition may be due to clinal variation in their exposure to N deposition. Another study observed opposite effects of N addition on percent N and C: N ratios compared to the response of the two northern populations in our study [44]. Pivovaroff et al. [44] used 2.5 times more N (50 kg N ha^-1^ yr^-1^) in their multiyear fertilized plots compared to high N (20 kg N ha^-1^ yr^-1^) treatments in our study. This could have contributed to the contrasting results for some of the populations in our study. Differences in plant trait responses to N addition could also be attributed to local adaption of populations to variation in N deposition exposure but plants were grown in the greenhouse and field for 24 months before measuring plant traits to minimize these effects. Furthermore, N addition could have also interacted with other abiotic conditions such as drought, contributing to the variation among population responses to N addition. Although it is difficult to reach clear conclusions with a relatively small number of populations in our study, our findings nevertheless suggest that constitutive clinal variation in plant traits could mediate clinal plasticity in response to N addition.

N addition increased arthropod abundance to a similar extent among all plant populations, but had no effect on arthropod richness, evenness, and diversity. Arthropod abundance tracked the consistent increase in plant biomass with N addition. N addition increased plant biomass and arthropod abundance but had no effect on arthropod density, suggesting that N addition did not significantly affect plant quality or resistance to herbivores. Even though N addition altered plant traits (i.e. percent N and C: N ratios) potentially altering plant quality, arthropod density did not respond to changes in these plant traits. These findings contrast with our past study that showed a northward cline of increasing arthropod density [8], suggesting that plant population variation influenced plant traits, cascading to higher trophic levels [12,45]. These contrasting findings could be attributed to the potential interactive effect of N addition and other abiotic conditions (i.e. drought) that were not considered in our study and to low productivity at this site. Another study showed that plant performance decreased with drought in combination with N addition [42]. At this site, plants were more stressed and the initial plant size was smaller than plants in an adjacent site in the year prior common garden. It is possible that plants in an environment with low productivity in combination with drought conditions are not able to take advantage of available N.

In conclusion, we demonstrated plant population variation in response to a fundamental aspect of global change and results suggest that this variation could be predicted based on constitutive trait variation. At the same time, plant variation in trait plasticity need not imply population variation in the indirect effects of global change on plant-associated arthropod communities. In most ecosystems, plant-associated arthropod communities are an important component of biodiversity as well as a food source for many threatened insectivorous vertebrates that often guide habitat management in these ecosystems. Accordingly, additional studies that focus on the indirect effects of N addition and other aspects of global change on plant-associated arthropod communities are needed.

## Supporting information

S1 Fig Main and interactive effect of nitrogen addition on associated arthropod communities. Main and interactive effect of nitrogen addition on a) arthropod density (individuals*m^-3^ plant biomass), b) species richness, c) Pielou’s species evenness, and d) Shannon-Wiener diversity. Letters represent population: SD = San Diego, SM = Santa Monica, CAM = Cambria, SC = Santa Cruz, and GG = Golden Gate National Recreation Area. Numbers below letters represent population latitude. Note differences in scale of y-axis. Bars represent ±1SE. All show no significant main or interactive effects of P = plant population, N = nitrogen addition, or P x N = plant population x nitrogen addition.
(TIF)

S1 Table Main and interactive effect of plant population and nitrogen deposition on arthropod measures. Dependence of arthropod density, species richness, Pielou’s species evenness (*J’*), and Shannon-Wiener diversity (*H’*) on population, N addition, and population x N addition interactions. Significant results are shown in bold.
(PDF)

S1 Dataset Plant trait and associated arthropod data. (XLSX)
