# Supplementary figures and images for: A test for clinal variation in Artemisia californica and associated arthropod responses to nitrogen addition
Source: PLoS One. 2018 Feb 1;13(2):e0191997. doi: 10.1371/journal.pone.0191997 (PMC5794083; doi:10.1371/journal.pone.0191997)

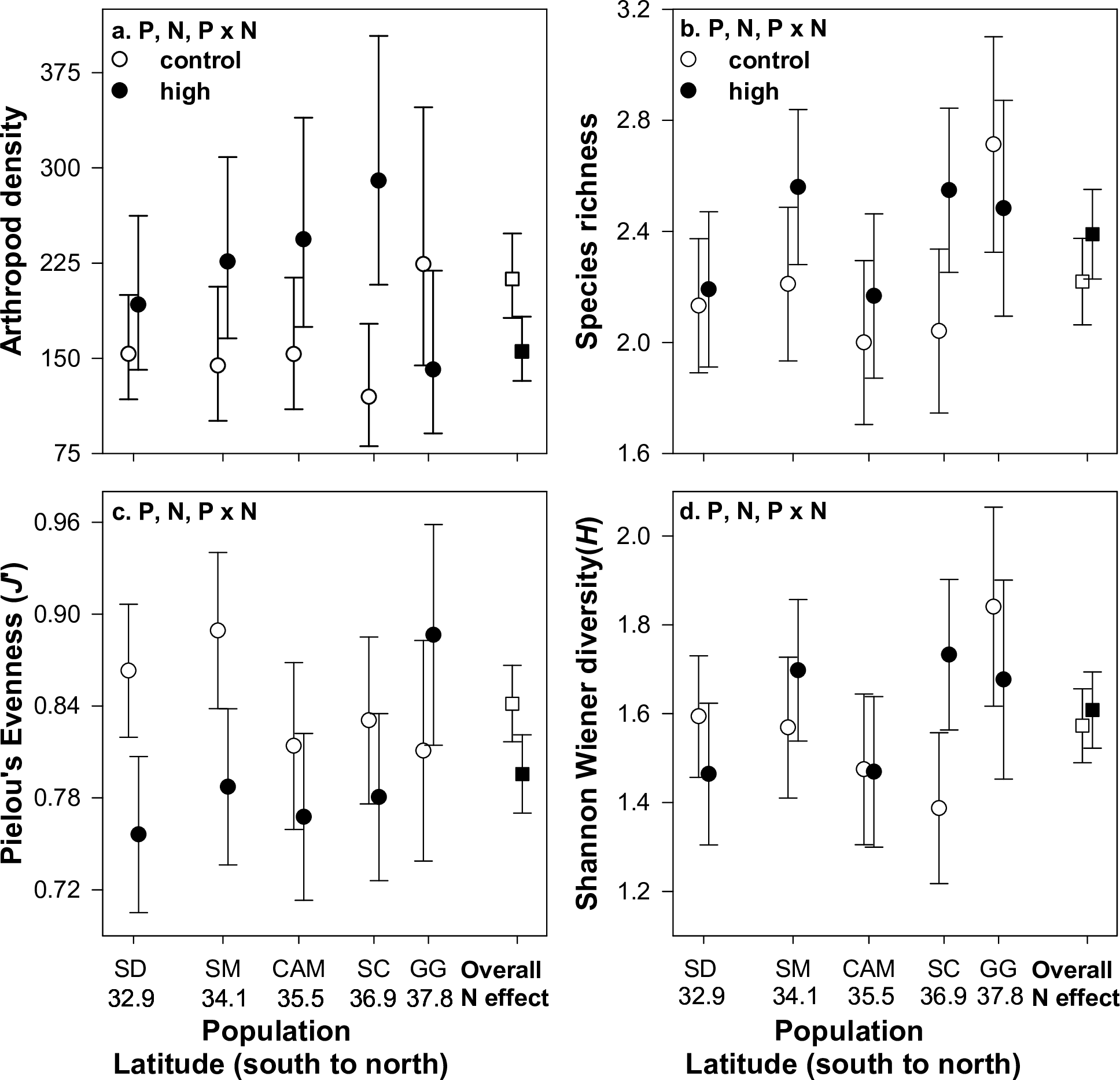

Supplement: S1 Fig — Main and interactive effect of nitrogen addition on a) arthropod density (individuals*m-3 plant biomass), b) species richness, c) Pielou’s species evenness, and d) Shannon-Wiener diversity. Letters represent population: SD = San Diego, SM = Santa Monica, CAM = Cambria, SC = Santa Cruz, and GG = Golden Gate National Recreation Area. Numbers below letters represent population latitude. Note differences in scale of y-axis. Bars represent ±1SE. All show no significant main or interactive effects of P = plant population, N = nitrogen addition, or P x N = plant population x nitrogen addition. (TIF) [file pone.0191997.s001.tif]
