# Supplementary material for: A test for clinal variation in Artemisia californica and associated arthropod responses to nitrogen addition
Source: PLoS One. 2018 Feb 1;13(2):e0191997. doi: 10.1371/journal.pone.0191997 (PMC5794083; doi:10.1371/journal.pone.0191997)
Supplement: S1 Table — Dependence of arthropod density, species richness, Pielou’s species evenness (J’), and Shannon-Wiener diversity (H’) on population, N addition, and population x N addition interactions. Significant results are shown in bold. (PDF) [file pone.0191997.s002.pdf]

S1 Table Main and interactive effect of plant population and nitrogen deposition on arthropod measures.

**Variable**	**Population** F_{DF} (P-value)	**Nitrogen** $F_{1,13}$ (P-value)	**Population x Nitrogen** F_{DF} (P-value)
Density	$0.04_{4,44}$ (0.9969)	1.93 (0.1879)	$0.75_{4,44}$ (0.5655)
Species richness	$0.79_{4,48}$ (0.5394)	0.58 (0.4601)	$0.40_{4,48}$ (0.8106)
Pielou’s Species Evenness	$0.33_{4,48}$ (0.8550)	1.66 (0.2207)	$0.66_{4,48}$ (0.6210)
Shannon-Wiener diversity	$0.66_{4,48}$ (0.6226)	0.09 (0.7741)	$0.75_{4,48}$ (0.5610)

Dependence of arthropod density, species richness, Pielou’s species evenness (*J’*), and Shannon-Wiener diversity (*H’*) on population, N addition, and population x N addition interactions. Significant results are shown in bold
